# A predictive model and rapid multi-dynamic algorithm developed based on tumor-stroma percentage in gastric cancer: a retrospective, observational study

**DOI:** 10.1093/gastro/goae083

**Published:** 2024-10-11

**Authors:** Yitian Xu, Yan Yang, Feichi Cheng, Zai Luo, Yuan Zhang, Pengshan Zhang, Jiahui Qiu, Zhengjun Qiu, Chen Huang

**Affiliations:** Department of Gastrointestinal Surgery, Shanghai General Hospital, Shanghai Jiao Tong University School of Medicine, Hongkou District, Shanghai, P. R. China; Department of Gastrointestinal Surgery, Shanghai General Hospital, Shanghai Jiao Tong University School of Medicine, Hongkou District, Shanghai, P. R. China; Department of Gastrointestinal Surgery, Shanghai General Hospital, Shanghai Jiao Tong University School of Medicine, Hongkou District, Shanghai, P. R. China; Department of Gastrointestinal Surgery, First Affiliated Hospital of Bengbu Medical College, Bengbu, Anhui, P. R. China; Department of Gastrointestinal Surgery, Shanghai General Hospital, Shanghai Jiao Tong University School of Medicine, Hongkou District, Shanghai, P. R. China; Department of Gastrointestinal Surgery, Shanghai General Hospital, Shanghai Jiao Tong University School of Medicine, Hongkou District, Shanghai, P. R. China; Department of Gastrointestinal Surgery, Shanghai General Hospital, Shanghai Jiao Tong University School of Medicine, Hongkou District, Shanghai, P. R. China; Department of Gastrointestinal Surgery, Shanghai General Hospital, Shanghai Jiao Tong University School of Medicine, Hongkou District, Shanghai, P. R. China; Department of Gastrointestinal Surgery, Shanghai General Hospital, Shanghai Jiao Tong University School of Medicine, Hongkou District, Shanghai, P. R. China; Department of Gastrointestinal Surgery, Shanghai General Hospital, Shanghai Jiao Tong University School of Medicine, Hongkou District, Shanghai, P. R. China

**Keywords:** gastric cancer, tumor-stroma percentage, nomogram, algorithm

## Abstract

**Background:**

Tumor-stroma percentage (TSP) is a prognostic risk factor in numerous solid tumors. Despite this, the prognostic significance of TSP in gastric cancer (GC) remains underexplored. Through the development of a personalized predictive model and a semi-automatic identification system, our study aimed to fully unlock the predictive potential of TSP in GC.

**Methods:**

We screened GC patients from Shanghai General Hospital (SGH) between 2012 and 2019 to develop and validate a nomogram. Univariate and multivariate Cox proportional hazards regression analyses were employed to identify independent prognostic factors influencing the prognosis for GC patients. The nomogram was further validated externally by using a cohort from Bengbu Medical College (BMC). All patients underwent radical gastrectomy, with those diagnosed with locally advanced GC receiving adjuvant chemotherapy. The primary outcome measured was overall survival (OS). The semi-automatic identification of the TSP was achieved through a computer-aided detection (CAD) system, denoted as TSP-cad, while TSP identified by pathologists was labeled as TSP-visual.

**Results:**

A total of 813 GC patients from SGH and 59 from BMC were enrolled in our study. TSP-visual was identified as an adverse prognostic factor for OS in GC and was found to be associated with pathological Tumor Node Metastasis staging system (pTNM) stage, T stage, N stage, perineural invasion (PNI), lymphovascular invasion (LVI), TSP-visual, tumor size, and other factors. Multivariate Cox regression using the training cohort revealed that TSP-visual (hazard ratio [HR], 2.042; 95% confidential interval [CI], 1.485–2.806; *P *<* *0.001), N stage (HR, 2.136; 95% CI, 1.343–3.397; *P *=* *0.010), PNI (HR , 1.791; 95% CI, 1.270–2.526; *P *=* *0.001), and LVI (HR, 1.482; 95% CI, 1.021–2.152; *P *=* *0.039) were independent predictors. These factors were incorporated into a novel nomogram, which exhibited strong predictive accuracy for 5-year OS in the training, internal validation, and external validation cohorts (area under the curve = 0.744, 0.759, and 0.854, respectively). The decision curve analysis of the nomogram and concordance indexes across the three cohorts outperformed the traditional pTNM (*P *<* *0.05). Additionally, TSP-cad assessment using a rapid multi-dynamic algorithm demonstrated good agreement with TSP-visual.

**Conclusions:**

The novel nomogram based on TSP could effectively identify individuals at risk of a poor prognosis among patients with GC. TSP-cad is anticipated to enhance the evaluation process of TSP.

## Introduction

Gastric cancer (GC) stands as one of the most prevalent cancers globally. According to GLOBOCAN, its incidence and mortality rates were ranked as the fifth and fourth highest worldwide in 2020 [1]. Surgical resection, typically complemented by adjuvant chemotherapy, represents the primary treatment approach for stage II and III GC, contributing to enhanced therapeutic outcomes [2]. In addition, immunotherapy and targeted therapy are available for suitable GC patients [3, 4]. Nevertheless, the prediction of recurrence and metastasis following gastrectomy remains challenging, leading to a poor prognosis for some patients [5]. In the case of peritoneal recurrence, the average survival time is <12 months [6]. Early prognosis prediction for GC patients enables the utilization of personalized perioperative adjuvant therapy to lower the risk of recurrence and metastasis. The identification of risk factors for poor survival in gastric cancer patients is crucial.

The interaction between tumor cells and their surrounding microenvironment has acquired increasing attention in recent years [7]. The tumor microenvironment consists of immune cells, fibroblasts, endothelial cells, and cytokines in the extracellular stroma. Tumorigenesis and progression are influenced not only by genetic factors, but also by the co-evolution of tumor cells with various components of the microenvironment, such as the extracellular stroma and immune cells. In fact, it can be viewed as an ecological disease characterized by dynamic interactions between malignant and non-malignant cells. The interplay between tumor cells and interstitial components plays a crucial role in determining the malignant biological behaviors related to tumor growth, invasion, and metastasis [8]. Tumor-stroma percentage (TSP) is a novel pathological index to parameterize the relationship between tumor cells and tumor stroma, which can be assessed by microscopic examination of hematoxylin and eosin (H&E)-stained tissue samples [9]. TSP is defined as the percentage of tumor stroma in selected areas of the most invasive part of the primary tumors [10]. A growing body of evidence emphasizes that TSP can effectively determine the prognosis for various solid tumors, such as colorectal, pancreatic, oropharyngeal, and breast cancers [11–14].

Previous studies on GC and TSP have demonstrated that high TSP is significantly correlated with an elevated 5-year mortality rate (hazard ratio [HR], 2.19; 95% confidence interval [CI], 1.69–2.85) [15]. Nevertheless, before TSP can be implemented in clinical settings, several challenges need to be addressed. Primarily, the majority of existing studies on TSP in GC are conducted at single centers [16, 17], highlighting the need for external cohorts to validate the accuracy and consistency of TSP in predicting patient outcomes. Second, due to the complexity and heterogeneity of the GC mechanism, relying solely on TSP assessment has limited effectiveness in predicting the prognosis of the disease. The construction of a predictive model that incorporates TSP and other prognostic factors is essential for enhanced prognostic accuracy. Finally, visual evaluation of H&E-stained histological images to determine TSP inevitably leads to a significant amount of workload and errors. Concordance of the assessment of TSP from different pathologists would also be poor. Therefore, we need a reliable semi-automatic method to enhance the competence of current treatment strategies to detect TSP in GC [18].

In an effort to address the aforementioned issues related to TSP, we placed our faith in the creation of a nomogram and the utilization of a previously developed rapid multi-dynamic algorithm [19] in this investigation. A nomogram is a graphical predictive tool used to assess the likelihood of events (such as mortality or recurrence) based on individual patient characteristics [20]. Through the utilization of our TSP-based nomogram, clinicians could promptly evaluate the overall survival (OS) for each GC patient, enabling the development of a tailored postoperative treatment strategy. The rapid multi-dynamic algorithm, grounded in supervised machine learning and pixel classification, had successfully facilitated the quick and consistent semi-automatic identification of TSP in GC patients.

By applying our nomogram and rapid multi-dynamic algorithm, it is ultimately intended to improve the prognostic significance of TSP, thereby facilitating the integration of TSP detection into clinical practice.

## Methods

### Study population

**Figure 1.**
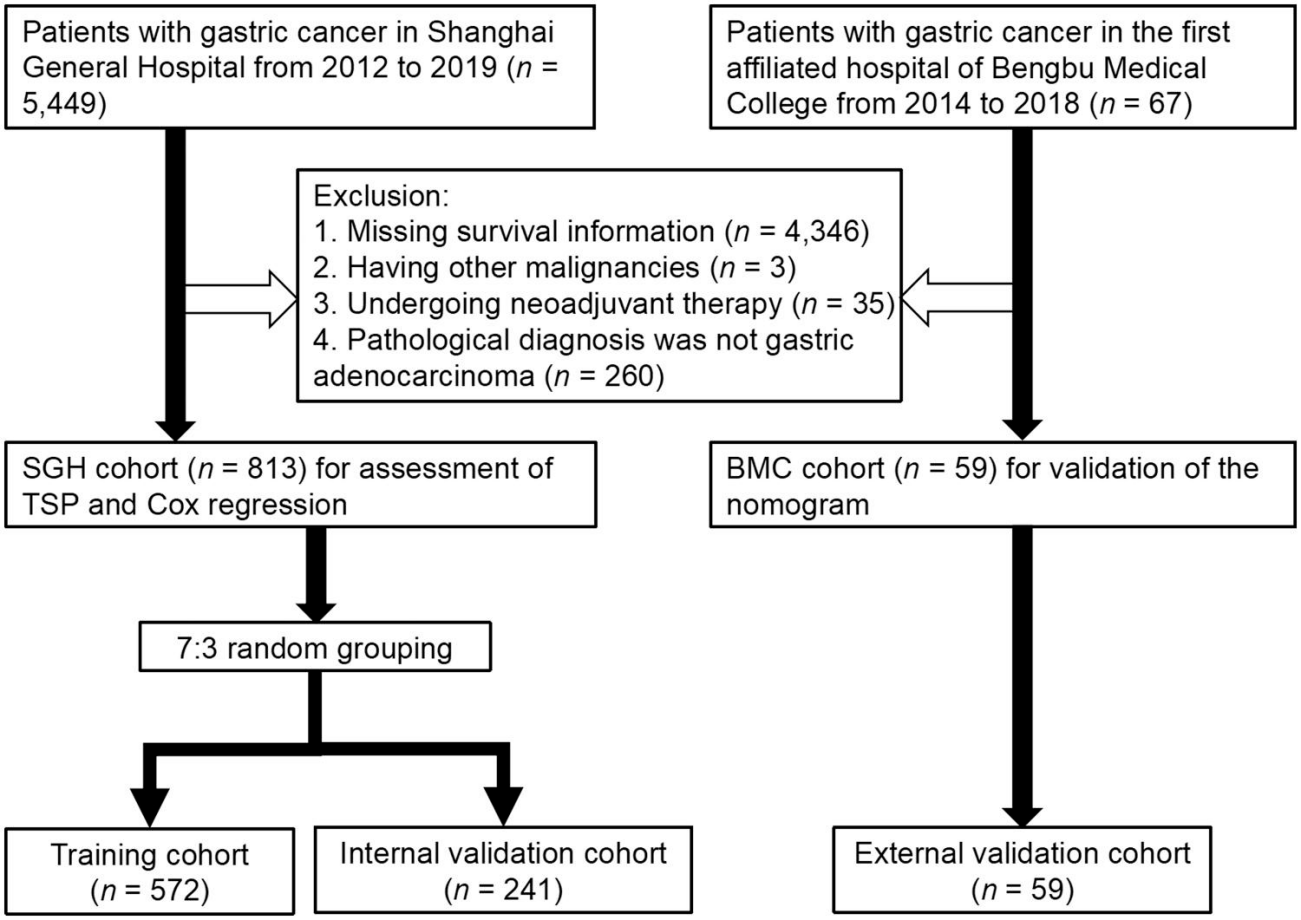
Flowchart of study sample selection.

This study retrospectively selected 813 patients who were diagnosed with GC and underwent radical resection at the Department of Gastrointestinal Surgery in Shanghai General Hospital (SGH), China between December 2012 and December 2019. The R function “createDataPartition” was used to split the entire SGH cohort into a training cohort (*n *=* *572) and an internal validation cohort (*n *=* *241) with a ratio of 7:3 through randomization. Figure 1 displays a flow chart illustrating the inclusion and exclusion criteria for the initial 5,449 cases from SGH. The inclusion criterion required that all patients had undergone gastrectomy in SGH between 2012 and 2019. The exclusion criteria for the large sample included patients with missing survival information, patients with other primary malignancies, patients who had received neoadjuvant therapy prior to surgical treatment, and patients who were not pathologically diagnosed with gastric adenocarcinoma. The diagnosis and subtype classification of GC were based on World Health Organization histological diagnostic criteria. The primary location of GC was based on the Japanese classification of gastric cancer (3rd edition). The study utilized the American Joint Committee on Cancer (AJCC) 8th Staging System to perform pathologic staging, assessing pathological Tumor Node Metastasis staging system (pTNM) stage, T stage, and N stage in this research. For each variable, we required duplicated entry by two trained professional pathologists. If any discrepancies were found, a third specialist would take part in discussions and make a final decision. This study was conducted in compliance with the Declaration of Helsinki and was approved by the Ethics Committee of Shanghai General Hospital (Approval number: 2021KSQ352).

A total of 59 consecutive patients who underwent radical resection for GC at the First Affiliated Hospital of Bengbu Medical College (BMC) between January 2014 and September 2018 were chosen as an independent cohort, following the same inclusion and exclusion criteria. This group of patients constituted the external validation cohort for this study.

### Assessment of TSP

In the SGH GC cohort, we used a KF-PRO series KF-PRO-120 digital section scanner to scan 5-μm-thick H&E-stained tissue sections to obtain high-resolution, 10× totally magnified digital images. Visual evaluation of TSP (TSP-visual) was assessed on digital images of H&E-stained sections of GC using the K-Viewer software application (http://www.kfbio.cn/download.php). In cases in which the patient had multiple lesions, we selected the most invasive section. The entire image of the GC tissue was viewed to identify the most invasive region and exported for further evaluation. Tumor cells were present on the north, south, east, and west boundaries of the selected area. All slides were double scored in a blinded fashion. The TSP-visual was independently scored by two pathologists and calculated (per 10-fold: 10%, 20%, 30%, and so on) per field. In cases in which a consensus could not be reached, a third pathologist was consulted for scoring and making a final decision. Images with blurring, extrusion, necro-inflammation, mucinous, or glassy lesions were excluded from evaluation. TSP-visual was scored using the method developed by Mesker *et al.* [21] and Ahn *et al.* [22] (TSP of <50% was classified as low TSP and TSP of ≥50% was classified as high TSP). TSP-visual for the BMC GC cohort was determined by using a similar method.

**Figure 2.**
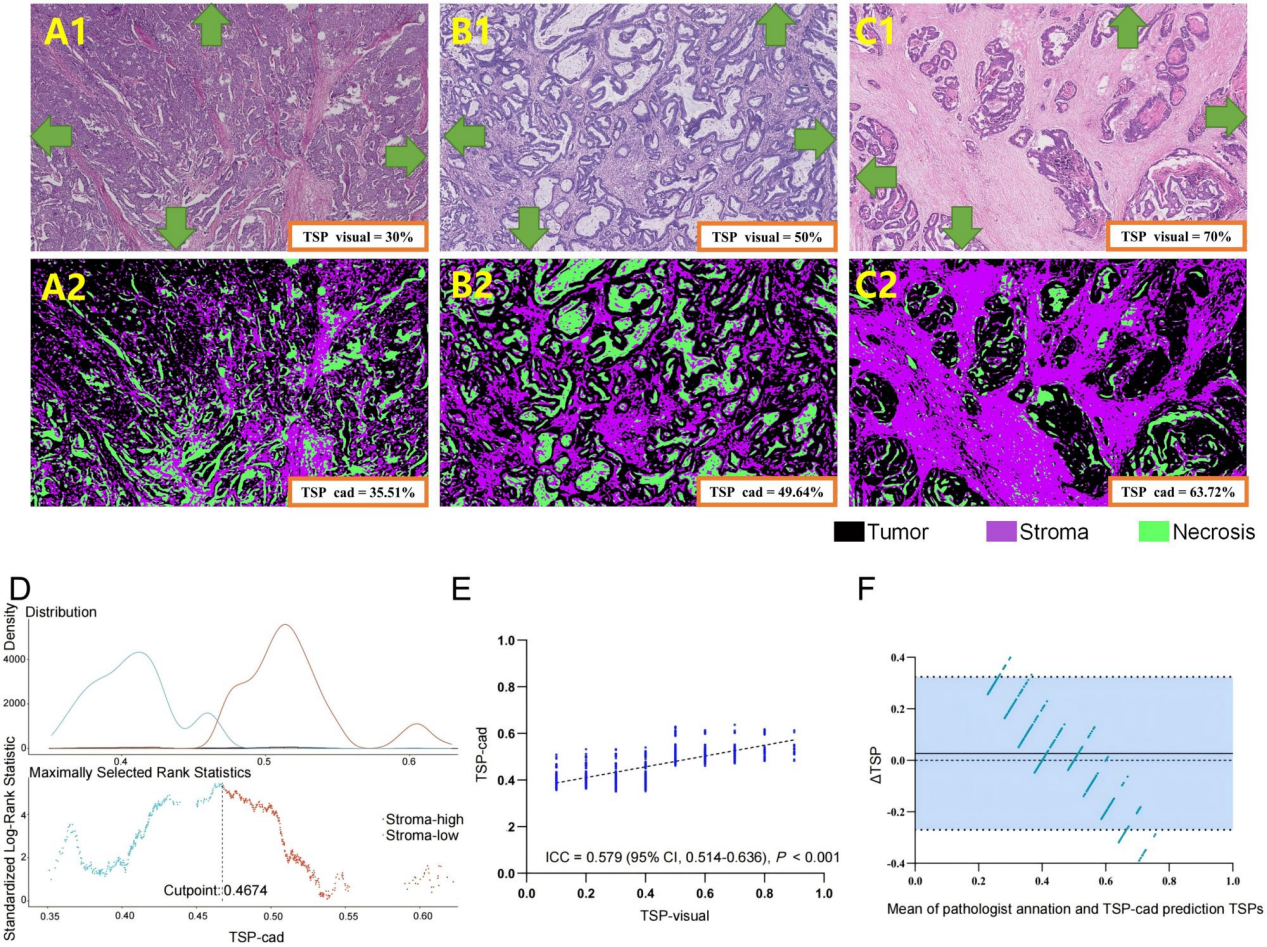
The assessment of TSP-visual and TSP-cad. Hematoxylin and eosin (H&E)-stained 5-μm paraffin sections were examined from the most invasive part of the primary gastric cancer. Tumor cells has to be present at all borders of the image field (north–east–south–west, corresponding arrows to indicate). Guideline for TSP-visual: (A1) stroma-low (TSP = 30%), (B1) stroma-moderate (50%), and (C1) stroma-high (70%). The regions with tissues segmented by using the rapid multi-dynamic algorithm for the calculation of TSP-cad: (A2) stroma-low (TSP-cad = 35.51%), (B2) stroma-moderate (TSP-cad = 49.64%), and (C2) stroma-high (TSP-cad = 63.72%). Please note that images were annotated using black for tumor, purple for stroma, and green for necrosis. (D) The cut-off determination: the optimal cut-off to categorize TSP-cad as stroma-low and stroma-high was determined by using the maximally selected rank statistics method. (E) Concordance between TSP-cad estimated by using the algorithm and TSP-visual estimated by the pathologist. (F) Bland–Altman plot for TSP estimation. The solid horizontal line is the mean, the dashed line is zero, and the shaded regions are 95% CIs. TSP = tumor-stroma percentage, CI = confidence interval.

Then, we processed the images by using a previously developed a rapid multi-dynamic algorithm based on supervised machine learning and pixel classification, the workflow of which was described in the Methods section of our previous research [19]. This computer-aided detection (CAD) system was used to analyse the grayscale images of tumor stroma. TSP-cad represented the percentage of tumor stroma as determined by the CAD system. Figure 2A–C shows a representative example. We defined the median as the threshold for TSP-cad (median) in the SGH GC queue. The optimal truncation value of TSP-cad (auto) was determined by using the maximum selective rank statistical method. The threshold of TSP-cad (50%) was defined by using the same method as that for TSP-visual.

### Predictors

Several inflammatory ratios of GC patients, identified from recent studies and commonly available in clinical practice [23, 24], were incorporated into this study. The parameters examined in our model development encompassed age (years), gender, histological type, tumor size, tumor site, grade, pTNM stage, T stage, N stage, perineural invasion (PNI), lymphovascular invasion (LVI), TSP, as well as tumor and inflammatory markers such as Ki-67, HER2, neutrophil-to-lymphocyte ratio (NLR), platelet-to-lymphocyte ratio (PLR), lymphocyte-to-monocyte ratio (LMR), and systemic immune-inflammation index (SII). Bias was minimized by prioritizing variables that could be objectively assessed and by excluding those with substantial missing data. For instance, histological differentiation grade was removed due to its subjectivity and high prevalence of missing values.

### Collection of survival information

The primary outcome of this study was OS, which was defined as the duration from the date of surgery to either death from any cause or the last follow-up, whichever occurred later. The surgery date was retrieved from electronic medical records, while survival status was determined through telephone follow-up. The initial follow-up took place 1 month post-surgery, with subsequent monitoring typically occurring at 3-month intervals for the first 2 years and then every 6 months. The study was censored in April 2022. During the final follow-up visit in January 2023, patients were reevaluated for OS.

### Statistical analysis

Statistical analysis was performed using IBM SPSS statistical software (Version 26). The construction and validation of the nomogram were carried out using R software version 4.3.1 (http://www.r-project.org/). Categorical data were compared by using the χ^2^ test. Prognostic factors for model construction were selected through univariate and multivariate Cox regression analyses. The nomogram and its calibration plots were generated using the R package “rms.” Receiver-operating characteristic (ROC) curves were plotted by using the R package “timeROC” to assess predictive performance. Concordance indexes (C-indexes) were calculated using the “Hmisc” package, and decision curve analyses (DCA) were conducted by using the “dcurves” package. Survival analyses were performed by using the Kaplan–Meier method. All tests were two-sided and *P*-values of <0.05 were considered statistically significant.

## Results

### Demographics and clinical characteristics in the SGH cohort

A total of 813 GC patients were statistically analysed following inclusion and exclusion criteria. Mesker *et al.* [21] and Ahn *et al.* [22] reported that the cut-off for TSP-visual was defined as 50%. In the whole group, 425 (52.3%) patients had low TSP-visual and 388 (47.7%) patients had high TSP-visual (Table 1). The median age at diagnosis was 67 years (range, 26–95 years). Finally, 573 (70.48%) males and 240 (29.52%) females were included in the SGH cohort. The number of patients with pTNM stage I, II, and III diseases were 223 (27.43%), 199 (24.48%), and 391 (48.09%), respectively. The numbers of patients with T1, T2, T3, and T4 diseases were 183 (22.51%), 110 (13.53%), 209 (25.71%), and 311 (38.25%), respectively; those of patients with N0, N1, N2, and N3 groups were 305 (37.52%), 132 (16.24%), 139 (17.10%), and 237 (29.15%), respectively. The majority of GC cases were classified as adenocarcinoma (85.85%). Additional clinicopathological and peripheral blood characteristics of GC patients are detailed in Table 1.

**Table 1.** Descriptive statistics for 813 patients with gastric cancer and comparison of characteristics according to TSP-visual

Variable	Training cohort			Internal validation cohort			
	Low TSP-visual (*n *=* *305)	High TSP-visual (*n *=* *267)	*P*-value	Low TSP-visual (*n *=* *120)	High TSP-visual (*n *=* *121)	*P*-value	***P* value** ^a^
**Age (years), *n* (%)**							
≤45	12 (3.9)	11 (4.1)	0.748	7 (5.8)	3 (2.5)	0.440	0.883
46–60	72 (23.6)	53 (19.9)		23 (19.2)	29 (24.0)		
61–75	161 (52.8)	146 (54.7)		65 (54.2)	68 (56.2)		
≥76	60 (19.7)	57 (21.3)		25 (20.8)	21 (17.4)		
**Gender, *n* (%)**							
Male	211 (69.2)	190 (71.2)	0.606	85 (70.8)	87 (71.9)	0.855	0.586
Female	94 (30.8)	77 (28.8)		35 (29.2)	34 (28.1)		
**Histological type, *n* (%)**							
Adenocarcinoma	268 (87.9)	228 (85.4)	0.681	99 (82.5)	103 (85.1)	0.852	0.912
Signet ring cell carcinoma	26 (8.5)	27 (10.1)		16 (13.3)	14 (11.6)		
Others	11 (3.6)	12 (4.5)		5 (4.2)	4 (3.3)		
**Tumor size (cm), *n* (%)**							
<5	200 (65.6)	150 (56.2)	**0.021**	74 (61.7)	69 (57.0)	0.463	**0.019**
≥5	105 (34.4)	117 (43.8)		46 (38.3)	52 (43.0)		
**Tumor site, *n* (%)**							
Proximal	64 (21.0)	87 (32.6)	**0.005**	20 (16.7)	28 (23.1)	0.452	**0.004**
Middle	93 (30.5)	62 (23.2)		37 (30.8)	34 (28.1)		
Distal	148 (48.5)	118 (44.2)		63 (52.5)	59 (48.8)		
**pTNM stage, *n* (%)**							
I	105 (34.4)	57 (21.3)	**<0.001**	34 (28.3)	27 (22.3)	**0.006**	**<0.001**
II	90 (29.5)	55 (20.6)		35 (29.2)	19 (15.7)		
III	110 (36.1)	155 (58.1)		51 (42.5)	75 (62.0)		
**T stage, *n* (%)**							
T1	76 (24.9)	55 (20.6)	**0.007**	30 (25.0)	22 (18.2)	0.468	**0.012**
T2	52 (17.0)	24 (9.0)		16 (13.3)	18 (14.9)		
T3	76 (24.9)	74 (27.7)		31 (25.8)	28 (23.1)		
T4	101 (33.1)	114 (42.7)		43 (35.8)	53 (43.8)		
**N stage, *n* (%)**							
N0	154 (50.5)	74 (27.7)	**<0.001**	46 (38.3)	31 (25.6)	**0.001**	**<0.001**
N1	52 (17.0)	38 (14.2)		28 (23.3)	14 (11.6)		
N2	50 (16.4)	53 (19.9)		16 (13.3)	20 (16.5)		
N3	49 (16.1)	102 (38.2)		30 (25.0)	56 (46.3)		
**PNI, *n* (%)**							
Negative	175 (61.2)	108 (42.5)	**<0.001**	53 (46.5)	47 (40.5)	0.361	**<0.001**
Positive	111 (38.8)	146 (57.5)		61 (53.5)	69 (59.5)		
NA	19	13		6	5		
**LVI, *n* (%)**							
Negative	151 (53.4)	91 (36.8)	**<0.001**	58 (51.8)	37 (32.7)	**0.004**	**<0.001**
Positive	132 (46.6)	156 (63.2)		54 (48.2)	76 (67.3)		
NA	22	20		8	8		
**Ki-67, *n* (%)**							
Negative	39 (13.3)	27 (10.7)	0.362	21 (18.3)	14 (12.5)	0.230	0.161
Positive	255 (86.7)	225 (89.3)		94 (81.7)	98 (87.5)		
NA	11	15		5	9		
**HER2, *n* (%)**							
Negative	198 (73.3)	171 (71.3)	0.600	81 (78.6)	75 (73.5)	0.391	0.386
Positive	72 (26.7)	69 (28.7)		22 (21.4)	27 (26.5)		
NA	35	27		17	19		
**NLR, *n* (%)**							
<2.00	267 (88.1)	232 (87.2)	0.744	109 (90.8)	107 (88.4)	0.541	0.585
≥2.00	36 (11.9)	34 (12.8)		11 (9.2)	14 (11.6)		
NA	2	1		/	/		
**PLR, *n* (%)**							
<245.73	245 (80.6)	196 (73.7)	**0.049**	92 (76.7)	100 (82.6)	0.249	0.312
≥245.73	59 (19.4)	70 (26.3)		28 (23.3)	21 (17.4)		
NA	1	1		–	–		
**LMR, *n* (%)**							
<2.73	173 (56.9)	167 (63.3)	0.124	77 (64.2)	77 (63.6)	0.932	0.180
≥2.73	131 (43.1)	97 (36.7)		43 (35.8)	44 (36.4)		
NA	1	3		–	–		
**SII, *n* (%)**							
<506.55	208 (68.6)	180 (67.7)	0.803	75 (62.5)	80 (66.1)	0.558	0.857
≥506.55	95 (31.4)	86 (32.3)		45 (37.5)	41 (33.9)		
NA	2	1		–	–		

TSP = tumor-stroma percentage, pTNM = pathological Tumor Node Metastasis staging system, PNI = perineural invasion, LVI = lymphovascular invasion, HER2 = human epidermal growth factor receptor 2, NLR = neutrophil-to-lymphocyte ratio, PLR = platelet-to-lymphocyte ratio, LMR = lymphocyte-to-monocyte ratio, SII = systemic immune-inflammation index.

a Chi-square *P*-values for the association between TSP-visual and the other characteristics in the entire SGH cohort.

### The correlation between TSP-visual and the other clinical characteristics

For the subsequent survival analyses and construction of a predictive model, the entire SGH cohort were randomly divided into a training cohort of 572 patients and an internal validation cohort of 241 patients at a ratio of 7:3. There were no statistical differences in all parameters between the training and internal validation cohorts. The results of χ^2^ tests displayed that higher TSP-visual levels were associated with more advanced pTNM stage, more advanced N stage, and more likely positive LVI no matter whether it was the training cohort, the internal validation cohort, or the entire SGH cohort (*P *<* *0.05 for all). Meanwhile, tumor size, tumor site, T stage, and PNI significantly differed between patients with low TSP-visual and high TSP-visual in both the training cohort and the entire SGH cohort (*P *<* *0.05 for all). Details are displayed in Table 1 and Supplementary Figure S1. These results suggested that GC patients with high TSP-visual exhibited more unfavorable clinical characteristics, indicating potential adverse clinical outcomes

### Univariate and multivariate Cox regression analysis

To evaluate the prognostic significance of TSP-visual and identify prognostic factors, univariate and multivariate Cox regression analyses were conducted in the training cohort. The univariate analysis revealed that high TSP-visual was a significant risk factor for OS (HR, 2.413; 95% CI, 1.812–3.213, *P *<* *0.001) (Table 2). The univariate analysis of the other clinical characteristics showed that age, tumor size, tumor site, pTNM stage, T stage, N stage, PNI, LVI, Ki-67, NLR, PLR, and LMR were significantly associated with OS (*P *<* *0.05 for all). The multivariate Cox analysis displayed that N stage, LVI, PNI, and TSP-visual were independent risk factors for OS in GC patients (*P *=* *0.010, *P *=* *0.001, *P *=* *0.039, and *P *<* *0.001, respectively) (Table 2).

**Table 2.** Univariate and multivariate Cox regression analysis of variables in the training cohort

Variable	Univariate analysis			Multivariate analysis		
	HR	95% CI	*P*-value	HR	95% CI	*P*-value
**Age (years)**			**0.018**			
≤45	–	1	–			
46–60	1.090	0.481–2.470	0.837			
61–75	1.502	0.699–3.227	0.297			
≥76	2.060	0.940–4.513	0.071			
**Gender**						
Male	–	1	–			
Female	0.755	0.554–1.028	0.074			
**Tumor size (cm)**						
<5	–	1	–			
≥5	1.814	1.381–2.383	**<0.001**			
**Histological type**			0.608			
Adenocarcinoma	–	1	–			
Signet ring cell carcinoma	1.035	0.645–1.661	0.886			
Others	0.666	0.294–1.505	0.328			
**Tumor site**			**0.001**			
Proximal	–	1	–			
Middle	0.525	0.367–0.752	**<0.001**			
Distal	0.608	0.443–0.836	**0.002**			
**pTNM stage**			**<0.001**			
I	–	1	–			
II	2.173	1.338–3.531	**0.002**			
III	4.217	2.751–6.464	**<0.001**			
**T stage**			**<0.001**			
T1	–	1	–			
T2	2.321	1.288–4.180	**0.005**			
T3	2.677	1.622–4.420	**<0.001**			
T4	3.862	2.385–6.253	**<0.001**			
**N stage**			**<0.001**			**0.010**
N0	–	1	–	–	1	–
N1	2.069	1.300–3.294	**0.002**	1.415	0.820–2.441	0.213
N2	2.839	1.868–4.316	**<0.001**	1.757	1.067–2.893	**0.027**
N3	4.183	2.895–6.043	**<0.001**	2.136	1.343–3.397	**0.001**
**PNI**						
Negative	–	1	–	–	1	–
Positive	2.801	2.085–3.763	**<0.001**	1.791	1.270–2.526	**0.001**
**LVI**						
Negative	–	1	–	–	1	–
Positive	2.695	1.968–3.691	**<0.001**	1.482	1.021–2.152	**0.039**
**Ki-67**						
Negative	–	1	–			
Positive	1.872	1.104–3.174	**0.020**			
**HER2**						
Negative	–	1	–			
Positive	1.123	0.823–1.531	0.465			
**NLR**						
<3.8	–	1	–			
≥3.8	1.539	1.060–2.235	**0.023**			
**PLR**						
<189.9	–	1	–			
≥189.9	1.402	1.035–1.901	**0.029**			
**LMR**						
<5.2	–	1	–			
≥5.2	0.641	0.478–0.859	**0.003**			
**SII**						
<572.6	–	1	–			
≥572.6	1.187	0.889–1.584	0.245			
**TSP-visual**						
Low	–	1	–	–	1	–
High	2.413	1.812–3.213	**<0.001**	2.042	1.485–2.806	**<0.001**

HR = hazard ratio, 95% CI = 95% confidential interval, TSP = tumor-stroma percentage, pTNM = pathological Tumor Node Metastasis staging system, PNI = perineural invasion, LVI = lymphovascular invasion, HER2 = human epidermal growth factor receptor 2, NLR = neutrophil-to-lymphocyte ratio, PLR = platelet-to-lymphocyte ratio, LMR = lymphocyte-to-monocyte ratio, SII = systemic immune-inflammation index.

### Construction and validation of the nomogram for OS

**Figure 3.**
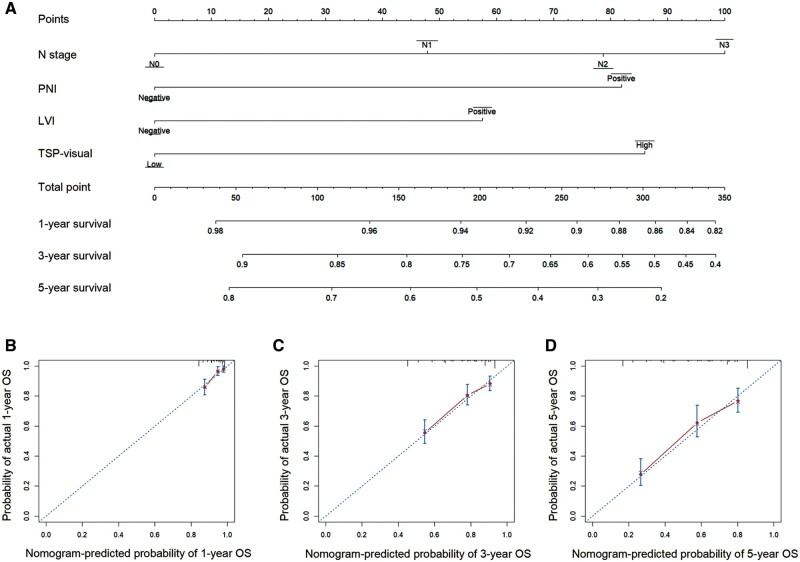
A novel nomogram and its calibration plots. A novel nomogram including TSP to predict 1-, 3-, and 5-year overall survival for gastric cancer patients (A) and the corresponding calibration curves of 1- (B), 3- (C), and 5-year overall survival (D). TSP = tumor-stroma percentage.

**Figure 4.**
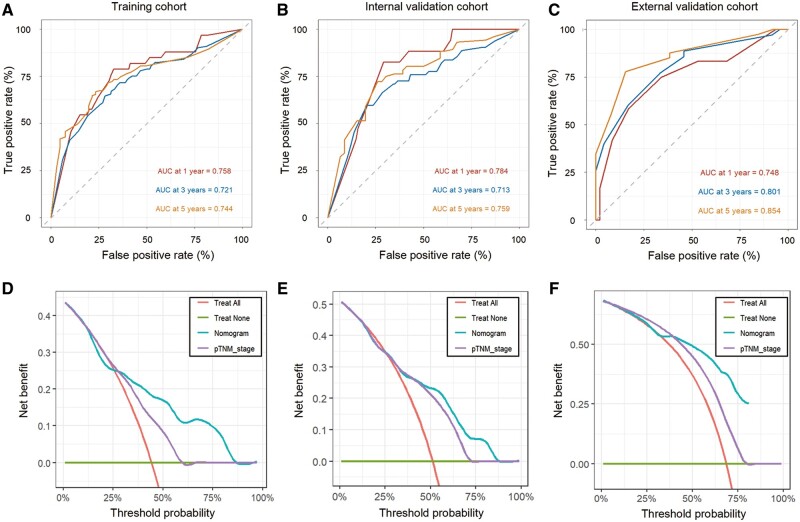
Receiver-operating characteristic curves and decision curve analysis. Receiver-operating characteristic analysis of the nomogram for 1-, 3-, and 5-year overall survival in the training cohort (A), internal validation cohort (B), and external validation cohort (C). Decision curve analyses of the clinical efficacy of the nomogram compared with the 8th edition of the American Joint Committee on Cancer pTNM classification in the training cohort (D), internal validation cohort (E), and external validation cohort (F).

Building upon the results of the multivariate Cox analysis, the combination of N stage, PNI, LVI, and TSP-visual was utilized to construct a personalized nomogram aimed at forecasting the OS likelihood of patients with GC (Figure 3A). The calibration plots demonstrated the strong agreement between the observed and predicted OS outcomes (Figure 3B–D). ROC curves were utilized to assess the predictive accuracy of our nomogram model for OS in GC patients (Figure 4A–C). In the training cohort, the area under the curve values (AUCs) for 1-, 3-, and 5-year OS were 0.758, 0.721, and 0.744, respectively. In the internal validation cohort, the AUCs for 1-, 3-, and 5-year OS were 0.784, 0.713, and 0.759, respectively. Subsequently, we further validated the predictive performance of the nomogram model in the BMC cohort. As shown in Supplementary Table S1, patients in the BMC cohort were younger and presented at a more advanced stage than those in the SGH cohort; however, the percentage of negative PNI and LVI was higher in the BMC cohort than in the SGH cohort. The AUCs for 1-, 3-, and 5-year OS in the external validation cohort were 0.748, 0.801, and 0.854, respectively, which further confirmed the accuracy of the nomogram. All of the AUC values exceeded 0.7, indicating that the novel nomogram possessed sufficient classification ability [25].

### Comparison between the nomogram and pTNM stage

Pathological stage is widely regarded as the most reliable prognostic indicator for GC [26]. Therefore, we conducted a comparative analysis between the predictive capabilities of the novel nomogram and the conventional pTNM staging system. Harrell’s C-index was utilized to assess the accuracy of the survival analysis model in ranking the risk levels among individuals. The C-indexes of the nomogram in the training, internal validation, and external validation cohorts (0.712, 0.709, and 0.738, respectively) were significantly higher than those of the pTNM stage (0.649, 0.664, and 0.596) (*P *<* *0.05 for all), which indicated that the nomogram could predict the OS of GC patients more accurately than the pTNM stage (Table 3). DCA was employed to evaluate the effectiveness of the predictive models in guiding clinical decision-making. Figure 4D and E shows the DCA curves for pTNM staging and the nomogram across the training, internal validation, and external validation cohorts. The DCA results indicated that the nomogram provided a greater net benefit than pTNM staging in all cohorts, underscoring the enhanced clinical utility of our nomogram over the pTNM stage. In summary, our nomogram demonstrated superior predictive performance in forecasting the OS of GC patients included in this study.

**Table 3.** Comparison of prediction accuracy between nomogram and pTNM stage

Variable	C-index	95% CI	*P*-value
**Training cohort**			**<0.001**
Nomogram	0.712	0.671–0.753	
pTNM stage	0.649	0.612–0.686	
**Internal validation cohort**			**0.021**
Nomogram	0.709	0.651–0.767	
pTNM stage	0.664	0.615–0.713	
**External validation cohort**			**0.012**
Nomogram	0.738	0.658–0.818	
pTNM stage	0.596	0.510–0.682	

C-index = concordance index, 95% CI = 95% confidential interval, pTNM = pathological Tumor Node Metastasis staging system.

### The assessment of TSP-cad

Three different TSP-cad cut-off values were chosen for comparison: 50%, the median, and the optimal truncation value (auto). The optimal truncation value (auto) was identified as 46.74% by using the maximally selected rank statistics method (Figure 2D). After classification, 491 (60.4%) patients were rated as low TSP-cad (50%) and 322 (39.6%) patients were rated as high TSP-cad (50%); 406 (49.9%) patients were rated as low TSP-cad (median) and 407 (50.1%) patients were rated as high TSP-cad (median); 376 (46.2%) patients were rated as low TSP-cad (auto) and 437 (53.8%) patients were rated as high TSP-cad (auto).

**Figure 5.**
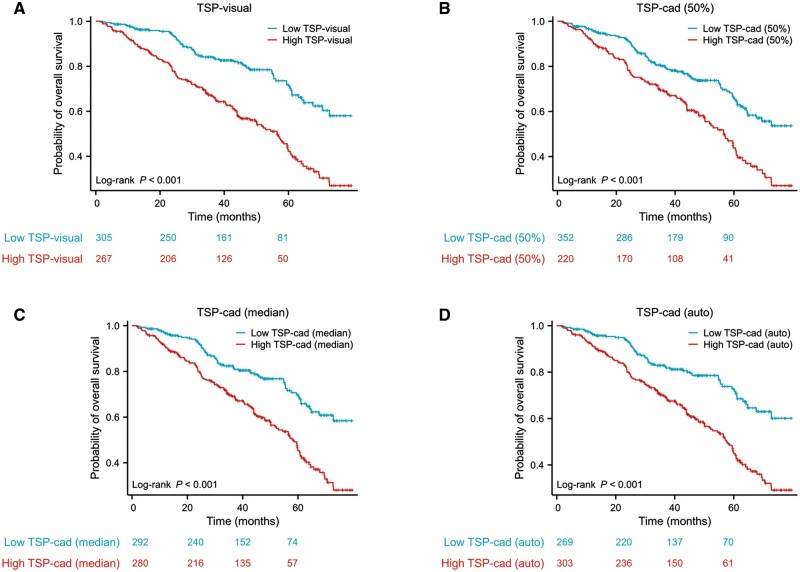
Kaplan–Meier analysis according to various TSP parameters. Kaplan–Meier analysis estimates the overall survival for gastric cancer patients in the training cohort according to TSP-visual (A), TSP-cad (50%) (B), TSP-cad (median) (C), and TSP-cad (auto) (D). TSP = tumor-stroma percentage.

Cox regression analysis of TSP-cad in the training cohort showed that TSP-cad (50%) (HR, 1.890; 95% CI, 1.439–2.484), TSP-cad (median) (HR, 2.090; 95% CI, 1.571–2.780), and TSP-cad (auto) (HR, 2.208; 95% CI, 1.642–2.970) all had a significant negative correlation with OS (*P *<* *0.05 for all). Kaplan–Meier analysis also proved that each TSP parameter was a valuable risk factor for the OS of GC patients (all log-rank *P *<* *0.001) (Figure 5A–D). Subsequently, we substituted TSP-visual with TSP-cad in the nomogram. ROC analysis revealed that the AUCs of the predictive models that integrated TSP-cad consistently exceeded 0.7, mirroring the outcomes of the original model (Supplementary Figure S2). Hence, TSP-cad (50%), TSP-cad (median), and TSP-cad (auto) for patients with low and high values were statistically different, with OS favoring patients with low TSP-cad. These results were the same as those for TSP-visual.

The Cohen’s Kappa coefficient between the two pathologists was 0.576 after dichotomizing TSP-visual according to a 50% cut-off (Supplementary Table S2). The Cohen’s Kappa coefficient between TSP-visual and TSP-cad (50%) was 0.717. Consistency was improved slightly (κ = 0.875) when using the median value (47.65%) as the cut-off for TSP-cad (median). When using the value (46.74%) obtained by using the maximally selected rank statistics method as the cut-off for TSP-cad (auto), the kappa coefficient between TSP-visual and TSP-cad (auto) was equal to 0.865 (Supplementary Table S2). A good agreement occurred between TSP-visual and TSP-cad (intraclass correlation coefficient [ICC] = 0.579; 95% CI, 0.514–0.636). A Bland–Altman plot showed good agreement between TSP predicted by using the rapid multi-dynamic algorithm and annotation by the pathologist. The mean difference was 0.0271 (95% CI, −0.2698 to 0.3240) (Figure 2E and F). As a result, there was good agreement between TSP-cad given by using the CAD system and TSP-visual evaluated by the pathologists.

## Discussion

In this study, we assessed the prognostic significance of the postoperative pathological indicator TSP and developed and validated a novel nomogram using data from the SGH and BMC cohorts. Furthermore, we attempted to achieve semi-automatic identification of TSP by using the CAD system. We found that patients exhibiting high TSP demonstrated a worse prognosis as indicated by using H&E-staining. Following univariate and multivariate Cox regression analyses, we developed a predictive nomogram incorporating TSP-visual, N stage, PNI, and LVI, demonstrating robust predictive accuracy and consistency. Finally, we demonstrated that TSP-cad had the potential to replace TSP-visual in our prediction model.

Our results indicated that GC patients with high TSP-visual tended to present with a more advanced pTNM stage, higher T stage and N stage, and an increased likelihood of perineural and lymphovascular invasion compared with those with low TSP-visual. These observations were similar to previous reports in Bokyung Ahn’s research [22]. The predictive value of TSP in determining OS was notably strong given that both Kaplan–Meier analysis and Cox regression analysis proved that TSP-visual was capable of risk stratification. Furthermore, we identified a significant association between high TSP-visual levels and proximal GC (*P *=* *0.004). Previous research had shown that patients with proximal GC had notably lower 1-, 3-, and 5-year OS rates than those with distal GC [27]. High TSP-visual might be one of the reasons for the poor prognosis for patients with proximal GC. It is plausible that elevated TSP-visual levels could contribute to the poor prognosis observed in patients with proximal GC. Given the current dearth of research in this area, our future investigations will focus on conducting subgroup analyses of patients with proximal GC to elucidate the relationship between TSP and tumor location.

The 8th edition of the AJCC pTNM staging system is superior to the 7th edition in terms of homogeneity, discriminatory ability, and monotonicity of gradients [28]. This updated pTNM staging system is one of the most frequently included prognostic factors and could discriminate patients with poor prognosis irrespective of country and ethnicity [29]. In many studies that incorporated multiple clinical parameters, pTNM staging turned out to be an independent risk factor for poor survival [30, 31]. However, the pTNM staging system has its limitations, as it exhibits homogeneity in prognostic prediction and only moderate predictive capacity [32]. In our study, the pTNM stage was not identified as an independent risk factor based on the results of multivariate Cox regression analysis (*P *>* *0.05), despite demonstrating significant performance in univariate analysis (*P *<* *0.001). As an alternative approach, we developed and validated a novel nomogram that integrates N stage, PNI, LVI, and TSP-visual to improve prognostic accuracy. The comparisons between the nomogram and pTNM stage showed that our nomogram was superior to pTNM stage in predicting the OS of GC patients. Therefore, when pTNM staging cannot accurately predict the survival of GC patients, our nomogram is a good choice. Furthermore, the nomogram is a visualized chart model that can assist clinicians in making cancer predictions in a more user-friendly and efficient manner [33]. So far, many nomogram models have been established to predict the survival of GC patients based on pathological indicators. In the study by Wang *et al.* [34], their nomogram incorporated pathological factors such as LVI and metastatic lymph nodes, with a C-index of 0.743 (95% CI, 0.710–0.776). Another nomogram, developed by Chen *et al.* [35], integrated the N stage and a pathomics signature derived from various features of H&E-stained slides, demonstrating superior accuracy. Unlike prior studies, our nomogram introduces TSP for the first time to forecast the OS of GC patients. While there is room for enhancing predictive precision, our model boasts full internal and external validation and a reduced number of predictors for practical use, and eliminates the necessity for extra pathological tissue staining.

Due to variations in results that stem from visual assessments by pathologists, it is challenging to eliminate detection bias related to TSP. To mitigate the subjective detection bias, we re-assessed TSP in our GC dataset using a rapid multi-dynamic algorithm that was previously developed [19], which accurately quantified the percentage of stroma area, significantly improving the predictive accuracy of TSP in GC. In our study, the internal consistency between our two pathologists in visual assessment of TSP was low (κ = 0.576), whereas the TSP that was evaluated using the rapid multi-dynamic algorithm showed high agreement with the assessments made by pathologists (κ = 0.875, 0.717, and 0.865, respectively). In terms of prognostic value, both TSP-cad and TSP-visual demonstrated similar performance. Considering that the visual assessment of TSP required more manpower and was less stable, the rapid multi-dynamic algorithm that we developed has the potential to serve as a crucial component of a prognostic tool for GC, offering high accuracy and simplicity. Implementing the TSP assessment offers advantages over other potential biomarkers due to its ease of implementation, requiring less time and cost. Furthermore, the rapid multi-dynamic algorithm that we have developed enables the standardization and replication of TSP assessment on readily available H&E-stained histological images, thereby eliminating errors and reducing the workload associated with visual evaluation. This semi-automated workflow is well suited for clinical practice and has the potential to expedite the clinical application of TSP in predicting and guiding treatment decisions for GC. Overall, our study highlights TSP as a robust predictor of survival in GC patients, with its clinical utility further enhanced by the integration of the rapid multi-dynamic algorithm into routine practice.

A recent study using artificial intelligence (AI) to develop a model called MIL-GC for predicting the outcome of GC also yielded meaningful results [36]. Our study focused on the connection between the tumor stroma and tumor tissue in GC, while their study concentrated on the specific pathological histological features from the stomach tissue. Both of these studies used computer-aided technology and had an effect on predicting the OS of GC. Their model exhibited less accuracy (C-index = 0.657) in comparison with ours, but their study reminds us that, by combining TSP with the pathological histopathological features of GC tissues, we might be able to develop a more satisfactory prediction model.

In addition, the cut-offs for TSP-cad in GC (median and auto) were 46.74% and 47.65%, respectively. Interestingly, our TSP-cad cut-offs are similar to those reported in the literature [37], with both cut-offs below the 50% cut-off. Due to tumor heterogeneity, TSP varies considerably between patients with different tumors. For example, studies have shown that marked interstitial fibrosis is a key feature of pancreatic ductal adenocarcinoma [38], contributing to tumor progression and the relative resistance of tumor cells to chemotherapy [39]. Therefore, the cut-off values for TSP may not be consistent across various tumor types. It is reasonable to consider that the optimal cut-off value for GC may be lower than the previously assumed 50%.

This study implies that the prognostic significance of TSP could lead to improved treatment strategies. In clinical practice, clinicians can assess postoperative survival rates using TSP and our nomogram. If a GC patient’s prognosis is determined to be poor, more frequent follow-up visits and additional examinations, such as DNA testing, may be recommended. However, this study has the typical limitations associated with retrospective studies. First, we focused solely on OS and did not analyse the prognostic value of TSP in disease-free survival, which is also a crucial outcome event for GC prognosis. Second, we did not investigate other preoperative inflammatory markers, such as C-reactive protein, that are not routinely assessed. We did not detect microsatellite instability in the included patients, which could have also led to differential expression of inflammatory markers. Third, only patients who underwent radical surgery were included, leading to the exclusion of stage IV patients and a high proportion of stage III patients. This limitation suggests that the findings of this study may not be generalizable to non-surgical GC patients.

In future endeavors, our intention is to gather additional patient data from different medical facilities to enhance the validation of the innovative nomogram via a multicenter study. The inclusion of liquid biopsy markers such as circulating tumor cells and circulating free DNA could potentially enhance the predictive accuracy of this model. Additionally, we aim to advance the utilization of TSP-cad in clinical settings and investigate the association between proximal GC and TSP.

## Conclusions

TSP has proved to be a significant prognostic factor for GC patients. A novel nomogram that incorporates TSP-visual, N stage, PNI, and LVI was created and validated to identify individuals at risk of poor OS in GC patients. This tool offers clinicians an accurate and efficient method for early prediction, aiding in the development of personalized treatment strategies. Moreover, we assessed TSP-cad by utilizing a rapid multi-dynamic algorithm based on supervised machine learning and pixel classification. This CAD system demonstrated enhanced speed, simplicity, and objectivity.

## Supplementary Material

goae083_Supplementary_Data

## References

[1] Sung H , Ferlay J, Siegel RL et al Global cancer statistics 2020: GLOBOCAN estimates of incidence and mortality worldwide for 36 cancers in 185 countries. CA Cancer J Clin 2021;71:209–49. 33538338 10.3322/caac.21660

[2] Japanese Gastric Cancer Association. Japanese Gastric Cancer Treatment Guidelines 2021 (6th edition). Gastric Cancer 2023;26:1–25. 36342574 10.1007/s10120-022-01331-8 PMC9813208

[3] Zhao Q , Cao L, Guan L et al Immunotherapy for gastric cancer: dilemmas and prospect. Brief Funct Genomics 2019;18:107–12. 30388190 10.1093/bfgp/ely019

[4] Joshi SS , Badgwell BD. Current treatment and recent progress in gastric cancer. CA Cancer J Clin 2021;71:264–79. 33592120 10.3322/caac.21657 PMC9927927

[5] Coccolini F , Nardi M, Montori G et al Neoadjuvant chemotherapy in advanced gastric and esophago-gastric cancer. Meta-analysis of randomized trials. Int J Surg 2018;51:120–7. 29413875 10.1016/j.ijsu.2018.01.008

[6] Yoo CH , Noh SH, Shin DW et al Recurrence following curative resection for gastric carcinoma. Br J Surg 2000;87:236–42. 10671934 10.1046/j.1365-2168.2000.01360.x

[7] Hinshaw DC , Shevde LA. The Tumor Microenvironment Innately Modulates Cancer Progression. Cancer Res 2019;79:4557–66. 31350295 10.1158/0008-5472.CAN-18-3962 PMC6744958

[8] Vangangelt KMH , Green AR, Heemskerk IMF et al The prognostic value of the tumor-stroma ratio is most discriminative in patients with grade III or triple-negative breast cancer. Int J Cancer 2020;146:2296–304. 31901133 10.1002/ijc.32857 PMC7065011

[9] Almangush A , Alabi RO, Troiano G et al Clinical significance of tumor-stroma ratio in head and neck cancer: a systematic review and meta-analysis. BMC Cancer 2021;21:480. 33931044 10.1186/s12885-021-08222-8 PMC8086072

[10] van Pelt GW , Sandberg TP, Morreau H et al The tumour-stroma ratio in colon cancer: the biological role and its prognostic impact. Histopathology 2018;73:197–206. 29457843 10.1111/his.13489

[11] Leppänen J , Lindholm V, Isohookana J et al Tenascin C, fibronectin, and tumor-stroma ratio in pancreatic ductal adenocarcinoma. Pancreas 2019;48:43–8. 30451798 10.1097/MPA.0000000000001195 PMC6296849

[12] Gao J , Shen Z, Deng Z et al Impact of tumor-stroma ratio on the prognosis of colorectal cancer: a systematic review. Front Oncol 2021;11:738080. 34868930 10.3389/fonc.2021.738080 PMC8635241

[13] Almangush A , Jouhi L, Haglund C et al Tumor-stroma ratio is a promising prognostic classifier in oropharyngeal cancer. Hum Pathol 2023;136:16–24. 37001738 10.1016/j.humpath.2023.03.010

[14] Vangangelt KMH , Tollenaar LSA, van Pelt GW et al The prognostic value of tumor-stroma ratio in tumor-positive axillary lymph nodes of breast cancer patients. Int J Cancer 2018;143:3194–200. 29978463 10.1002/ijc.31658

[15] Kemi N , Eskuri M, Kauppila JH. Tumour-stroma ratio and 5-year mortality in gastric adenocarcinoma: a systematic review and meta-analysis. Sci Rep 2019;9:16018. 31690815 10.1038/s41598-019-52606-7 PMC6831590

[16] Kim EY , Abdul-Ghafar J, Chong Y et al Calculated tumor-associated neutrophils are associated with the tumor-stroma ratio and predict a poor prognosis in advanced gastric cancer. Biomedicines 2022;10:708. 35327509 10.3390/biomedicines10030708 PMC8945075

[17] Kemi N , Eskuri M, Herva A et al Tumour-stroma ratio and prognosis in gastric adenocarcinoma. Br J Cancer 2018;119:435–9. 30057407 10.1038/s41416-018-0202-y PMC6133938

[18] Geessink OGF , Baidoshvili A, Klaase JM et al Computer aided quantification of intratumoral stroma yields an independent prognosticator in rectal cancer. Cell Oncol (Dordr) 2019;42:331–41. 30825182 10.1007/s13402-019-00429-z PMC12994350

[19] Li T , Yu Z, Yang Y et al Rapid multi-dynamic algorithm for gray image analysis of the stroma percentage on colorectal cancer. J Cancer 2021;12:4561–73. 34149920 10.7150/jca.58887 PMC8210572

[20] Balachandran VP , Gonen M, Smith JJ et al Nomograms in oncology: more than meets the eye. Lancet Oncol 2015;16:e173–e180. 25846097 10.1016/S1470-2045(14)71116-7 PMC4465353

[21] Mesker WE , Junggeburt JMC, Szuhai K et al The carcinoma-stromal ratio of colon carcinoma is an independent factor for survival compared to lymph node status and tumor stage. Cell Oncol 2007;29:387–98. 17726261 10.1155/2007/175276 PMC4617992

[22] Ahn B , Chae YS, Kim CH et al Tumor microenvironmental factors have prognostic significances in advanced gastric cancer. APMIS 2018;126:814–21. 30264431 10.1111/apm.12889

[23] Xu Y , Zhang P, Luo Z et al A predictive nomogram developed and validated for gastric cancer patients with triple-negative tumor markers. Future Oncol 2024;20:919–34. 37920954 10.2217/fon-2023-0626

[24] Nechita VI , Al-Hajjar N, Moiş E et al Inflammatory ratios as predictors for tumor invasiveness, metastasis, resectability and early postoperative evolution in gastric cancer. Curr Oncol 2022;29:9242–54. 36547138 10.3390/curroncol29120724 PMC9776857

[25] George B , Seals S, Aban I. Survival analysis and regression models. J Nucl Cardiol 2014;21:686–94. 24810431 10.1007/s12350-014-9908-2 PMC4111957

[26] Li Z , Wang Y, Ying X et al Different prognostic implication of ypTNM stage and pTNM stage for gastric cancer: a propensity score-matched analysis. BMC Cancer 2019;19:80. 30651085 10.1186/s12885-019-5283-3 PMC6335703

[27] Xue J , Yang H, Huang S et al Comparison of the overall survival of proximal and distal gastric cancer after gastrectomy: a systematic review and meta-analysis. World J Surg Oncol 2021;19:17. 33468158 10.1186/s12957-021-02126-4 PMC7816301

[28] Ji X , Bu ZD, Yan Y et al The 8th edition of the American Joint Committee on Cancer tumor-node-metastasis staging system for gastric cancer is superior to the 7th edition: results from a Chinese mono-institutional study of 1663 patients. Gastric Cancer 2018;21:643–52. 29168120 10.1007/s10120-017-0779-5 PMC6002446

[29] Wang W , Sun Z, Deng J-Y et al A novel nomogram individually predicting disease-specific survival after D2 gastrectomy for advanced gastric cancer. Cancer Commun (Lond) 2018;38:23. 29764518 10.1186/s40880-018-0293-0 PMC5993138

[30] Zhu X , Zhou G, Ma M et al Clinicopathological analysis and prognostic assessment of TCN1 in patients with gastric cancer. Surg Innov 2022;29:557–65. 34549663 10.1177/15533506211045318

[31] Liu HL , Feng X, Tang MM et al Prognostic significance of preoperative lymphocyte to monocyte ratio in patients with signet ring gastric cancer. World J Gastrointest Surg 2023;15:1673–83. 37701703 10.4240/wjgs.v15.i8.1673 PMC10494583

[32] Fang C , Wang W, Deng JY et al Proposal and validation of a modified staging system to improve the prognosis predictive performance of the 8th AJCC/UICC pTNM staging system for gastric adenocarcinoma: a multicenter study with external validation. Cancer Commun (Lond) 2018;38:67. 30454049 10.1186/s40880-018-0337-5 PMC6245913

[33] Zhu J , Han Y, Ni W et al Nomogram-based survival predictions and treatment recommendations for locally advanced esophageal squamous cell carcinoma treated with upfront surgery. Cancers (Basel) 2022;14:5567. 36428660 10.3390/cancers14225567 PMC9688301

[34] Wang PL , Xiao FT, Gong BC et al A nomogram for predicting overall survival of gastric cancer patients with insufficient lymph nodes examined. J Gastrointest Surg 2017;21:947–56. 28349332 10.1007/s11605-017-3401-6

[35] Chen D , Fu M, Chi L et al Prognostic and predictive value of a pathomics signature in gastric cancer. Nat Commun 2022;13:6903. 36371443 10.1038/s41467-022-34703-w PMC9653436

[36] Huang B , Tian S, Zhan N et al Accurate diagnosis and prognosis prediction of gastric cancer using deep learning on digital pathological images: a retrospective multicentre study. EBioMedicine 2021;73:103631. 34678610 10.1016/j.ebiom.2021.103631 PMC8529077

[37] Peng C , Liu J, Yang G et al The tumor-stromal ratio as a strong prognosticator for advanced gastric cancer patients: proposal of a new TSNM staging system. J Gastroenterol 2018;53:606–17. 28815347 10.1007/s00535-017-1379-1 PMC5910462

[38] Leppänen J , Lindholm V, Isohookana J et al Tenascin C, fibronectin, and tumor-stroma ratio in pancreatic ductal adenocarcinoma. Pancreas 2019;48:43–8. 30451798 10.1097/MPA.0000000000001195 PMC6296849

[39] Mayer P , Jiang Y, Kuder TA et al Diffusion kurtosis imaging-a superior approach to assess tumor-stroma ratio in pancreatic ductal adenocarcinoma. Cancers (Basel) 2020;12:1656. 32580519 10.3390/cancers12061656 PMC7352692

