# Supplementary material for: A predictive model and rapid multi-dynamic algorithm developed based on tumor-stroma percentage in gastric cancer: a retrospective, observational study
Source: Gastroenterol Rep (Oxf). 2024 Oct 11;12:goae083. doi: 10.1093/gastro/goae083 (PMC11470210; doi:10.1093/gastro/goae083)
Supplement: goae083_Supplementary_Data [file goae083_supplementary_data.zip › 2024-017 Supplementary_materials.docx]

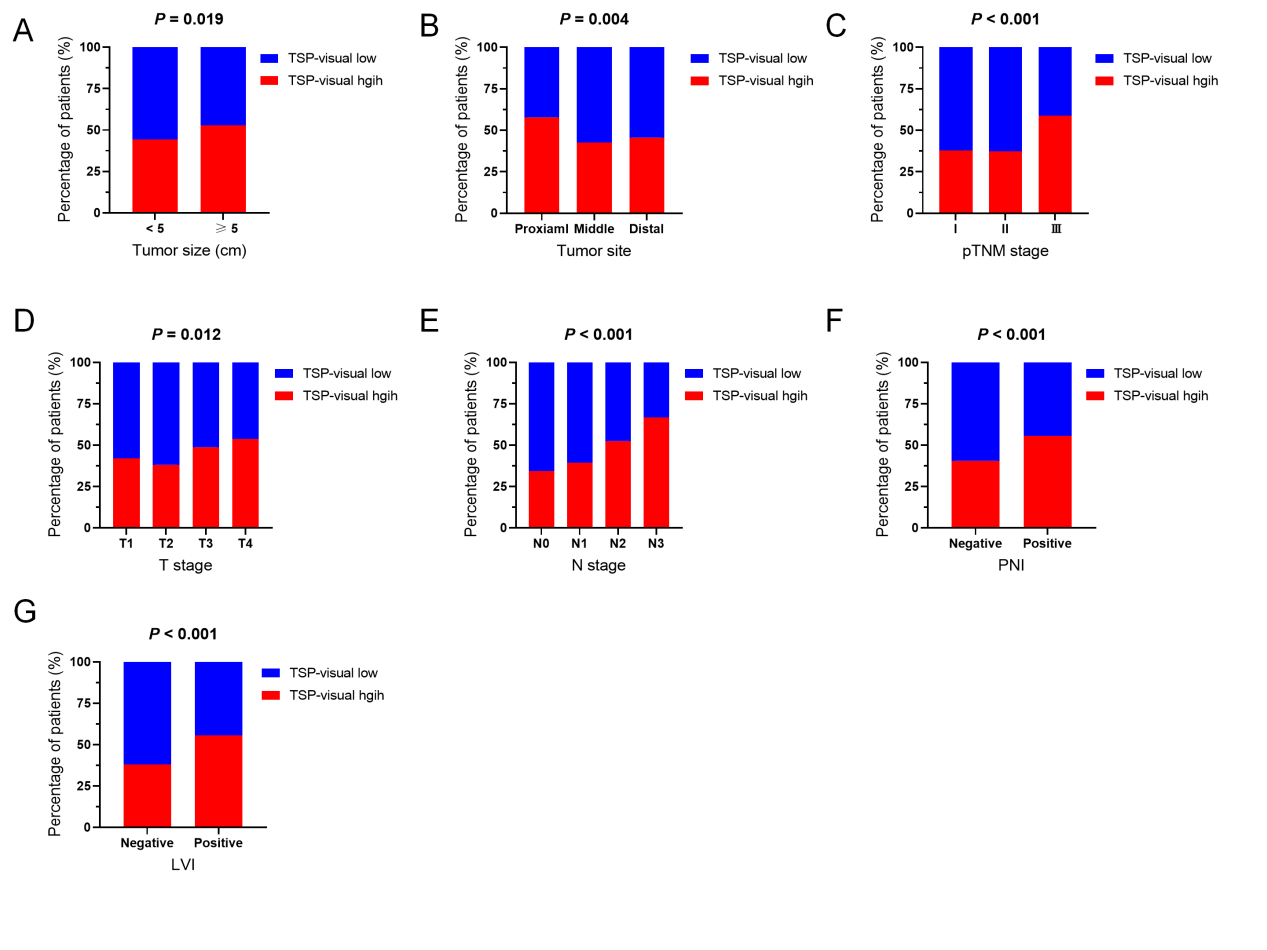


**Supplementary Figure S1.** Percentage of patients with low TSP-visual and high TSP-visual stratified by different category (pTNM stage, T stage, N stage, primary tumor location, tumor size, perineural invasion, and lymphovascular invasion) in the entire SGH cohort (*n* = 813).


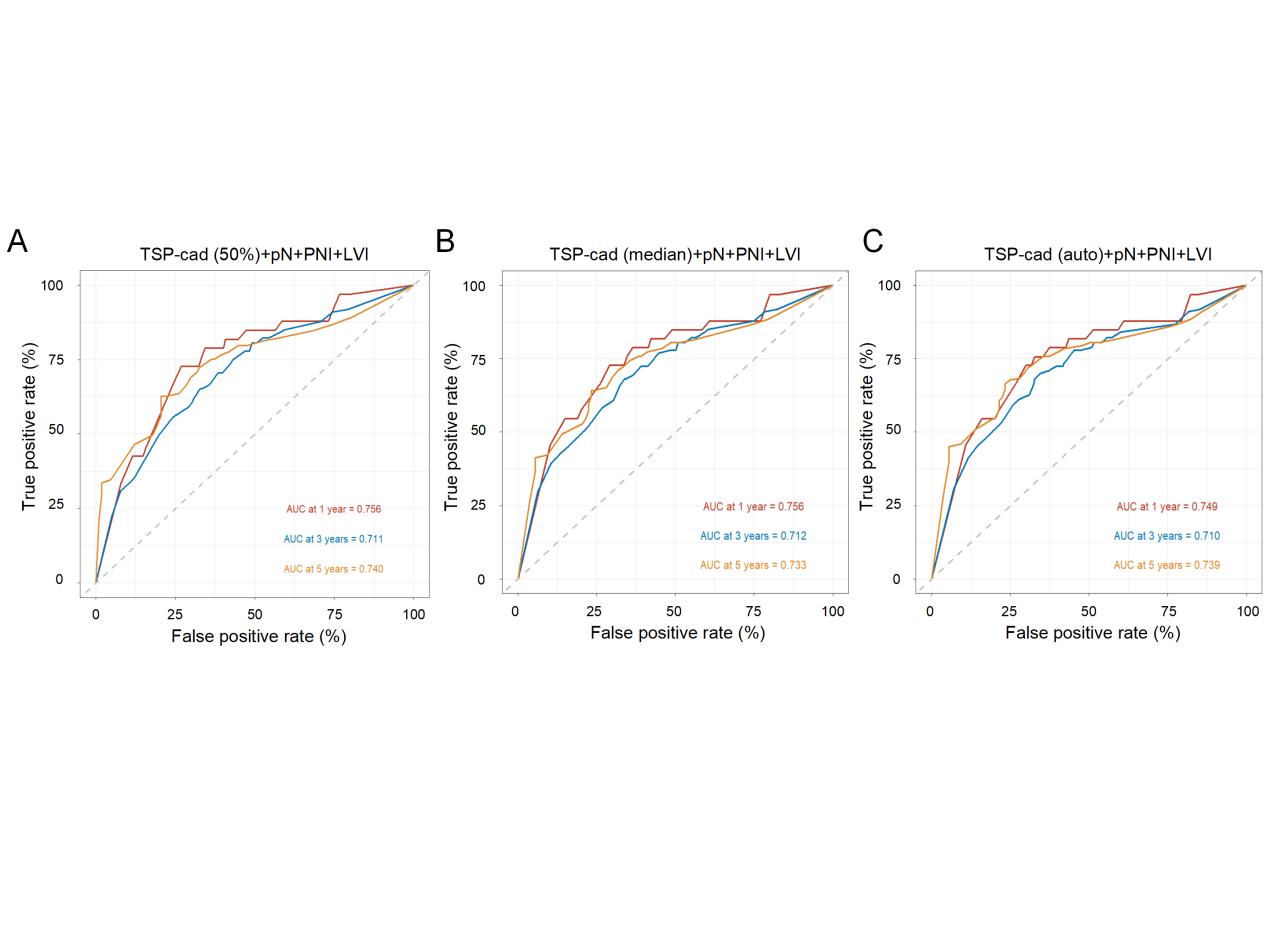


**Supplementary Figure S2.** Receiver operating characteristic curves of the predictive models whose TSP-visual was replaced with TSP-cad (50%) (A), TSP-cad (median) (B), or TSP-cad (auto) (C) in the training cohort.

| **Supplementary Table S1.** Comparison of the SGH and BMC cohorts | | | |
| --- | --- | --- | --- |
| **Variable** | **SGH cohort (*n* = 813)** | **BMC cohort (*n* = 59)** | ***P*** value |
| **Age (years), *n* (%)** |  |  | **<0.001** |
| ≤60 | 210 (25.8) | 29 (49.2) |  |
| ＞60 | 603 (74.2) | 30 (50.8) |  |
| **Gender, *n* (%)** |  |  | 0.663 |
| Male | 573 (70.5) | 40 (67.8) |  |
| Female | 240 (29.5) | 19 (32.2) |  |
| **Histological type, *n* (%)** |  |  | 0.450 |
| Adenocarcinoma | 698 (85.9) | 47 (79.7) |  |
| Signet ring cell carcinoma | 83 (10.2) | 9 (15.3) |  |
| others | 32 (3.9) | 3 (5.1) |  |
| **Tumor size (cm), *n* (%)** |  |  | 0.567 |
| ＜5 | 493 (60.6) | 38 (64.4) |  |
| ≥5 | 320 (39.4) | 21 (35.6) |  |
| **Tumor site, *n* (%)** |  |  | **<0.001** |
| Proxiaml | 199 (24.5) | 26 (44.1) |  |
| Middle | 226 (27.8) | 0 (0.0) |  |
| Distal | 388 (47.7) | 33 (55.9) |  |
| **pTNM stage, *n* (%)** |  |  | **0.003** |
| I | 223 (27.4%) | 5 (8.5%) |  |
| II | 199 (24.5%) | 22 (37.3%) |  |
| III | 391 (48.1%) | 32 (54.2%) |  |
| **T stage, *n* (%)** |  |  | **<0.001** |
| T1 | 183 (22.5) | 1 (1.7) |  |
| T2 | 110 (13.5) | 12 (20.3) |  |
| T3 | 209 (25.7) | 15 (25.4) |  |
| T4 | 311 (38.3) | 31 (52.5) |  |
| **N stage, *n* (%)** |  |  | 0.122 |
| N0 | 305 (37.5) | 24 (40.7) |  |
| N1 | 132 (16.2) | 13 (22.0) |  |
| N2 | 139 (17.1) | 13 (22.0) |  |
| N3 | 237 (29.2) | 9 (15.3) |  |
| **PNI, *n* (%)** |  |  | **<0.001** |
| Negative | 383 (49.7) | 58 (98.3) |  |
| Positive | 387 (50.3) | 1 (1.7) |  |
| NA | 43 | 0 |  |
| **LVI, *n* (%)** |  |  | **<0.001** |
| Negative | 337 (44.6) | 55 (93.2) |  |
| Positive | 418 (55.4) | 4 (6.8) |  |
| NA | 58 | 0 |  |
| **TSP-visual, *n* (%)** |  |  | 0.223 |
| Low | 425 (52.3) | 26 (44.1) |  |
| High | 388 (47.7) | 33 (55.9) |  |
| SGH, Shanghai General Hospital; BMC, Bengbu Medical College; pTNM, pathological Tumor Node Metastasis staging system; PNI, perineural invasion; LVI, lymphovascular invasion; TSP, tumor-stroma percentage. | | | |

**Supplementary Table S2.** Cross-tabulation of pathologist 1 versus pathologist 2, TSP-visual versus TSP-cad after dichotomisation in the SGH cohort

| κ = 0.576 | | Pathologis 2 | | | κ = 0.875 | | TSP-cad (median) | | |
| --- | --- | --- | --- | --- | --- | --- | --- | --- | --- |
|  |  | Low | High | Total |  |  | Low | High | Total |
| Pathologist 1 | Low | 342 | 83 | 425 | TSP-visual | Low | 390 | 35 | 425 |
|  | High | 89 | 299 | 388 |  | High | 16 | 372 | 388 |
|  | Total | 431 | 382 | 813 |  | Total | 406 | 407 | 813 |
| κ = 0.717 | | TSP-cad (50%) | | | κ = 0.865 | | TSP-cad (auto) | | |
|  |  | Low | High | Total |  |  | Low | High | Total |
| TSP-visual | Low | 401 | 24 | 425 | TSP-visual | Low | 373 | 52 | 425 |
|  | High | 90 | 298 | 388 |  | High | 3 | 385 | 388 |
|  | Total | 491 | 322 | 813 |  | Total | 376 | 437 | 813 |
| κ, Cohen’s Kappa coefficient; TSP, tumor-stroma percentage; CAD, computer-aided detection. | | | | | | | | | |
